# Genome-Wide Identification of β-Ketoacyl CoA Synthase Gene Family in Melon (*Cucumis melo* L.) and Its Expression Analysis in Autotoxicity, Saline-Alkali, and Microplastic Exposure Environments

**DOI:** 10.3390/cimb47030195

**Published:** 2025-03-16

**Authors:** Lizhen Zhang, Mingcheng Wang, Xianhuan Tang, Xinyue Yang, Zhizhong Zhang, Jinghua Wu

**Affiliations:** 1 College of Horticulture, Fujian Agriculture and Forestry University, Fuzhou 350002, China; zhanglizhen2025@163.com (L.Z.);; 2 Fujian Yongan Vegetable Science and Technology Backyard, Sanming 366000, China; 3 Key Laboratory of Ministry of Education for Genetics, Breeding and Multiple Utilization of Crops, Fuzhou 350002, China

**Keywords:** β-ketoacyl CoA synthase gene, cucurbits, molecular phylogeny, stress response gene, very-long-chain fatty acids

## Abstract

β-ketoacyl CoA synthase (KCS) is a key enzyme in the synthesis of long-chain fatty acids. It affects plant stress resistance by regulating the chain length of fatty acid elongation products, the wax deposition in plant epidermis, and the formation of suberization layers. Through a comprehensive, genome-wide analysis, we identified members of the melon *KCS* (*CmKCS*) family and characterized their sequence features, phylogenetic relationships, and expression profiles under three abiotic stress conditions, employing bioinformatics tools and methods. Fifteen *CmKCSs* were identified in the melon genome and found to be unevenly distributed across eight chromosomes. The subcellular localization of most members is located on the cytoplasmic membrane and chloroplasts. The *CmKCS* family amplifies its members in a tandem repeat manner, which is more closely related to the cucumber *KCS* and has similar gene functions. Subfamilies I, IV, and VI exhibit variations in conserved domain sequences, which may indicate specific functional differentiation. The promoter region harbors various cis-acting elements related to plant hormones and abiotic stress responses. Among these, the most abundant are elements responsive to abscisic acid, methyl jasmonate, salicylic acid, and anaerobic induction. *CmKCS5*, *CmKCS6*, *CmKCS10*, and *CmKCS12* showed high expression in autotoxicity, saline-alkali stress, and microplastic exposure environments. These four *CmKCSs* may play important roles in melon development and stress response. In conclusion, this study provides a comprehensive analysis of the *CmKCS* gene family, revealing its potential roles in melon’s response to abiotic stresses and laying a foundation for further functional characterization of these genes in stress tolerance mechanisms.

## 1. Introduction

Very-long-chain fatty acids (VLCFAs) are important precursors for the biosynthesis of wax and membrane lipids in plant tissues such as stems, leaves, fruits, and pollen. Meanwhile, lignin, another critical component of plant cell walls, is synthesized through the phenylpropanoid pathway. They play a crucial role in plant growth, development, and environmental adaptation [1,2,3]. Lignin and wax, composed of VLCFAs and their derivatives, such as alkanes, ketones, alcohols, aldehydes, and waxes, are important pathways for plants to cope with various stresses, such as pathogen invasion, ultraviolet radiation, low temperature, salinity, and drought. Plant cells synthesize various VLCFAs, and their components and synthesis pathways are complex. β-Ketoyl CoA synthase (*KCS*) is a key enzyme in the biosynthesis of VLCFAs. The elongation of VLCFAs occurs on the endoplasmic reticulum, and each elongation cycle consists of four steps: condensation, reduction, dehydroxylation, and re-reduction. *KCS* is the first rate-limiting enzyme in this catalytic process, involved in the condensation of Malonyl CoA and Long-chain acyl CoA, and also plays an important role in determining the chain length of fatty acid extension products [1]. The conserved domains of *KCS* protein include regions crucial for initiating chain reactions mediated by plant fatty acid synthase, as well as active elements involved in substrate binding, which are related to the substrate specificity of the *KCS* enzyme [4,5].

The first *KCS* was isolated from an *Arabidopsis thaliana* mutant lacking VLCFAs, which is involved in fatty acid metabolism during seed development [6]. Twenty-one members of *KCSs* were identified in *Arabidopsis*, and *KCS1*, *KCS2*, *KCS6*, *KCS9*, and *KCS20* were confirmed to be involved in the wax synthesis of the epidermis [7]. *KCS9* is primarily involved in the elongation of C22 and C24 fatty acids, while *KCS*6 catalyzes the elongation of C26 or longer carbon chains. The overexpression of *KCS6* significantly increases epidermal wax content in *Arabidopsis*, making it a key gene for wax synthesis [8]. In rice (*Oryza sativa* L.), plants overexpressing *KCS1* showed a significant increase in wax content in the leaf epidermis, decreased permeability, and significantly enhanced drought resistance without adverse effects on major agronomic traits [9]. In the *HvKCS1* mutant of *Hordeum vulgare* L., there were significant changes in the crystal structure and chemical composition of wax, with a significant decrease in primary alcohol content, which in turn affected the cuticle structure, epidermal barrier performance, and disease resistance of the plant [10].

Melon (*Cucumis melo* L.) belongs to the *Cucurbitaceae* family and is a perennial herbaceous plant with a unique flavor, short growth cycle, and high cultivation economic benefits. It is widely cultivated around the world, and its production environment often faces various unfavorable factors. For example, drought, salinity, and continuous cropping obstacles directly affect the yield and quality of melon, severely restricting the healthy development of melon production [11,12]. In addition, in recent years, facility cultivation has been widely utilized, and the residues of agricultural plastics continue to accumulate in farmland. The potential threat of new pollutants, such as microplastics, is also gradually emerging. In our preliminary research, we found that the synthesis of suberin and wax in melon roots was enhanced under autotoxicity and saline-alkali stress. The *KCS* was located at the center of the interaction network of differentially expressed genes and was closely related to root stress response [13]. At present, there is no comprehensive analysis of the *CmKCS* family.

Therefore, this study aims to: (1) comprehensively identify and characterize the *KCS* gene family in melon based on the whole genome sequence; (2) analyze their physicochemical properties, phylogenetic relationships, gene structures, and expression patterns to elucidate their roles in fatty acid metabolism and wax biosynthesis; and (3) investigate the expression levels of *CmKCS* under adverse environmental conditions, including autotoxicity, saline-alkali stress, and microplastic exposure, to explore their potential functions in melon stress resistance. We hypothesize that the *CmKCS* family plays a crucial role in melon’s adaptation to abiotic stresses by regulating the synthesis of protective compounds such as suberin and wax. The choice of melon as a study species is justified by its economic importance, susceptibility to multiple environmental stresses, and the lack of comprehensive research on the *KCS* family in this crop, which limits our understanding of its stress adaptation mechanisms.

## 2. Materials and Methods

### 2.1. Identification and Chromosome Localization of KCSs in Melon

The whole genome database of melon is obtained from Melon (DHL92) v 3.6.1 on the CuGenDB website (http://cucurbitgenomics.org/, accessed on 1 February 2025). The Hidden Markov Model (HMM) file of the *KCS* gene (PF08392) was downloaded from the Pfam database (http://pfam.xfam.org/, accessed on 1 February 2025). Using TBtools (v1.120) software for a local BLAST search, candidate gene sequences of the *CmKCS* family were obtained. The amino acid sequences of all members of the *Arabidopsis KCS* family were downloaded from the *Arabidopsis* TAIR database (https://www.arabidopsis.org/, accessed on 1 February 2025). The candidate gene protein sequences were further confirmed using the online software SMART (http://SMART.embl-heidelberg.de, accessed on 1 February 2025) and CDD website (https://www.ncbi.nlm.nih.gov/cdd, accessed on 1 February 2025) to exclude genes with incomplete functional domains. The genes containing the conserved domains ACP_Syn_SII_C (PF08541) and FAE1_CUT1_RppA (PF08392) were ultimately identified as members of the *CmKCS* family. The chromosome positional information of the *CmKCS* family member was obtained from the melon whole genome annotation file, which was downloaded from Melon (DHL92) 3.6.1. The visualization of results and chromosome localization maps were completed using the Sequence Toolkits option in TBtools software.

### 2.2. Physicochemical Properties and Subcellular Localization of CmKCS Member

The prediction of basic physicochemical properties, such as relative molecular weight, isoelectric point, amino acid quantity, and instability coefficient of *CmKCS* family members, was completed using the protein online analysis software ExPASY (https://web.expasy.org/protparam/, accessed on 1 February 2025). The subcellular localization prediction of *CmKCS* family members was completed through the online software WoLFPSORT (https://wolfpsort.hgc.jp/, accessed on 1 February 2025).

### 2.3. Systematic Evolution Analysis of the KCS Family

The protein sequence information of *Arabidopsis KCS* family members was obtained from the TAIR website (http://meme-suite.org/tools/meme, accessed on 5 February 2025). The protein sequence information of *KCS* family members in melon, watermelon, cucumber, winter melon, and Indian pumpkin was obtained from CuGenDB. The *KCS* protein sequence alignment of the above species was completed using MEGA7.0 software, and the construction of the phylogenetic tree was carried out using the maximum likelihood method (ML). The optimal model for constructing the ML phylogenetic tree of the *KCS* family is the JTT + G + F type, which was obtained through the model testing module in TBtools (V2.110). The Bootstrap parameter was set to 1000 times, the model was set to JTT + G + F type, and the rest of the parameters were set to the system default. The phylogenetic tree editing and beautification were completed through iTOL online software (https://itol.embl.de/, accessed on 5 February 2025).

### 2.4. Structure and Conserved Motifs Analysis of CmKCS Family Members

The structure information of *CmKCS* family members was extracted from the annotation file of the whole melon genome and visualized using the Gene Structure View function in TBtoolsV2.110 software. The analysis of conserved motifs in *CmKCS* family members was completed using the online software MEME (https://meme-suite.org/meme/). The output motif was set to 15, and other parameters were set to the system default values. The drawing of the phylogenetic tree, gene structure, and domain combination diagram of *CmKCS* family members was completed using TBtools (V2.110).

### 2.5. Collinearity Analysis of the KCS Family

The whole genome files of melon, watermelon, cucumber, Indian pumpkin, winter melon, and *Arabidopsis thaliana* were downloaded from CuGenDB and TAIR websites. The collinearity analysis and visualization of the *KCS* family within the melon genome and between *Arabidopsis*, watermelon, cucumber, Indian pumpkin, and winter melon genomes were analyzed using TBtools software.

### 2.6. Analysis of Cis-Acting Elements in the Promoter Region of CmKCSs

Sequence information for the 2000 bp range upstream of the transcription start site of 15 *CmKCS* family members was downloaded from the CuGenDB website. The homeotropic elements of the promoter region of *CmKCS* were obtained through the online website PlantCARE (http://bioinformatics.psb.ugent.be/webtools/plantcare/html/, accessed on 1 February 2025). The visualization of the results was completed using TBtools software.

### 2.7. Expression Analysis of CmKCSs in Three Abiotic Stress Environments

The *CmKCS* family member IDs were retrieved from three transcriptome databases of melon under abiotic stress (owned by our group), including autotoxicity (NCBI accession number SRP242941) [14], saline-alkali [15], and microplastic exposure environments [16]. Then, the FPKM (fragments per kilobase of exon model per million mapped fragments) values of *CmKCS* members under differential stress were obtained. The gene expression heatmap was drawn using TBtools (V2.110) software.

## 3. Results

### 3.1. Identification of CmKCS Family Members and Chromosome Localization

**Figure 1:**
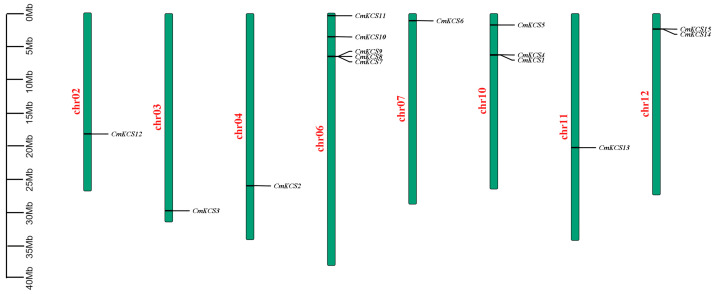
Chromosomal localization of *KCS*s in melon.

Fifteen members of the *CmKCS* family were identified in the melon whole genome and were named *CmKCS1* to *CmKCS15,* respectively. All 15 members contain the ACP_Syn_iII_C and FAE1_CUT1_RppA domains. Family members were distributed on 8 chromosomes (Figure 1). The maximum number of family members owned was chromosome 6, with 5 *CmKCS*s. Three and two family members were found on chromosomes 12 and 10, respectively. One member was found on each of chromosomes 2, 3, 4, 7, and 11.

### 3.2. CmKCS Structure and Encoded Protein Characteristics

The amino acid length of the protein encoded by *CmKCS* ranged from 469 aa (*CmKCS1*) to 588 aa (*CmKCS12*), with small differences in length between different members (Table 1). The molecular weight of *CmKCS* ranged from 33,861.82 Da (*CmKCS6*) to 66,670.98 Da (*CmKCS12*). The isoelectric point value of every *CmKCS* member was greater than 7, ranging from 7.23 (*CmKCS7*) to 9.24 (*CmKCS5*), indicating alkaline protein. The instability coefficient of *CmKCS* ranged from 28.74 (*CmKCS2*) to 47.98 (*CmKCS7*). Among them, 11 members with an instability coefficient less than 40 were stable proteins, and 4 members with an instability coefficient greater than 40 were unstable proteins. The proportion of *CmKCS* members located in the cytoplasmic membrane, chloroplasts, vacuoles, and cytoplasm was 46.67%, 33.33%, 13.33%, and 6.7%, respectively.

### 3.3. Phylogenetic Analysis of KCSs

**Figure 2:**
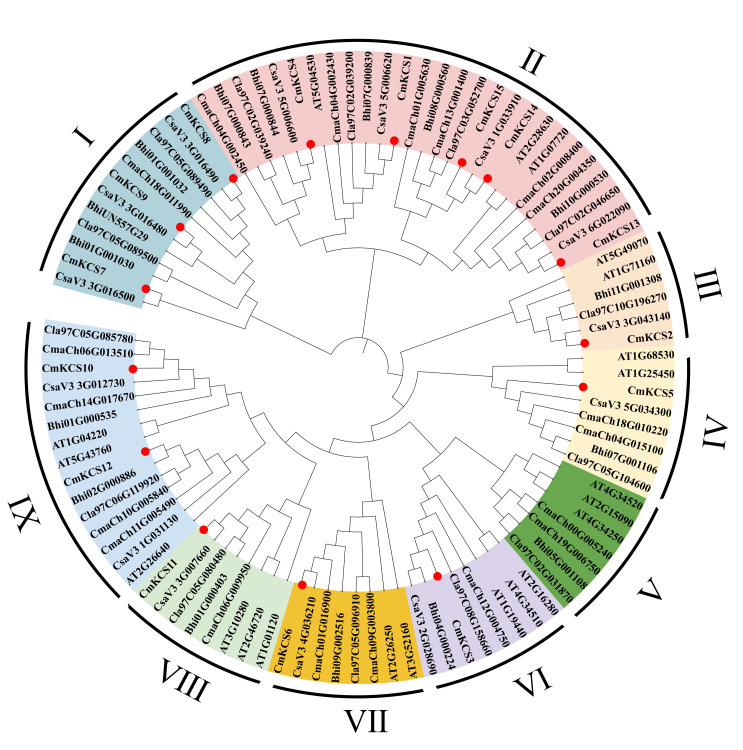
Phylogenetic tree of *KCSs* in melon and other plants.

A total of 99 *KCS* family members from 6 species were obtained from relevant websites and databases, including 15 from melon, 14 from watermelon, 14 from cucumber, 14 from Indian pumpkin, 16 from winter melon, and 21 from *Arabidopsis*. These genes were used to construct the phylogenetic tree of the *KCS* family, as shown in Figure 2. All *KCS* family members of 6 species can be classified into 9 subfamilies. The number of genes distributed in subfamilies I to IX was 12, 27, 6, 8, 7, 8, 8, and 15, respectively. Subfamily I only contained the *Cucurbitaceae KCS* family. Subfamily II contained the largest number of genes, and the most *KSC* family members of each species were distributed in this subfamily. The genetic relationship between *Arabidopsis KCS* and *Cucurbitaceae* plants was relatively distant, with 21 members distributed in subfamilies II to IX, each subfamily containing 2 or 3 members clustered individually. There were 3, 5, and 2 *CmKCS* members in subfamilies I, II, and IX, respectively. Except for subfamily V, all other subfamilies contained one *CmKCS*. The *KCS* number distributed in each subfamily of melon and cucumber was basically the same (except for subfamily II). The *KCS* members of the two were clustered in one branch as orthologs.

### 3.4. Gene Structure and Conserved Sequence of CmKCSs

**Figure 3:**
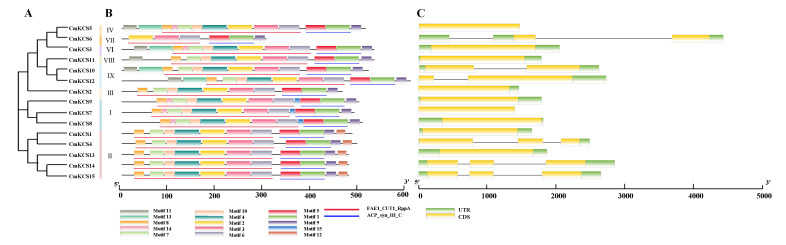
Phylogenetic relationships (**A**), motif composition (**B**), and gene structure (**C**) of *KCS*s in melon.

The gene structure of *CmKCS* is shown in Figure 3B. Family members generally contained 1–3 exons. There were 4 members with 2 introns, 2 members with 1 intron, and the remaining 9 members without introns. The gene structures of some members within the same subfamily were the same, such as *CmKCS3* and *CmKCS11*, *CmKCS14,* and *CmKCS15.* Fifteen conserved motifs were identified in the *CmKCS* protein (Figure 3C), and all members except *CmKCS*6 contained Motif1–Motif10. The domains corresponding to Motif1 and Motif5 were ACP_Syn_iII_C, while Motif2, Motif3, Motif4, Motif6, Motif7, Motif8, and Motif10 correspond to a part of domain FAE1_CUT1_RppA. Motif14 was also included in the FAE1_CUT1_RppA domain, appearing in I, IV, and VI subfamily member sequences, with a small amount of variation in the conserved sequence. Different clusters contained different conserved motifs, with Motif12 being a unique motif of the second subfamily and Motif15 being a unique motif of the first subfamily. Some conserved motifs were also shared among 2–3 subfamilies, such as Motif11 and Motif13, which were conserved motifs shared by subfamilies VI and IX, and Motif14, which was a conserved motif shared by subfamilies I, VI, and IV.

### 3.5. Collinearity Analysis of KCSs

**Figure 4:**
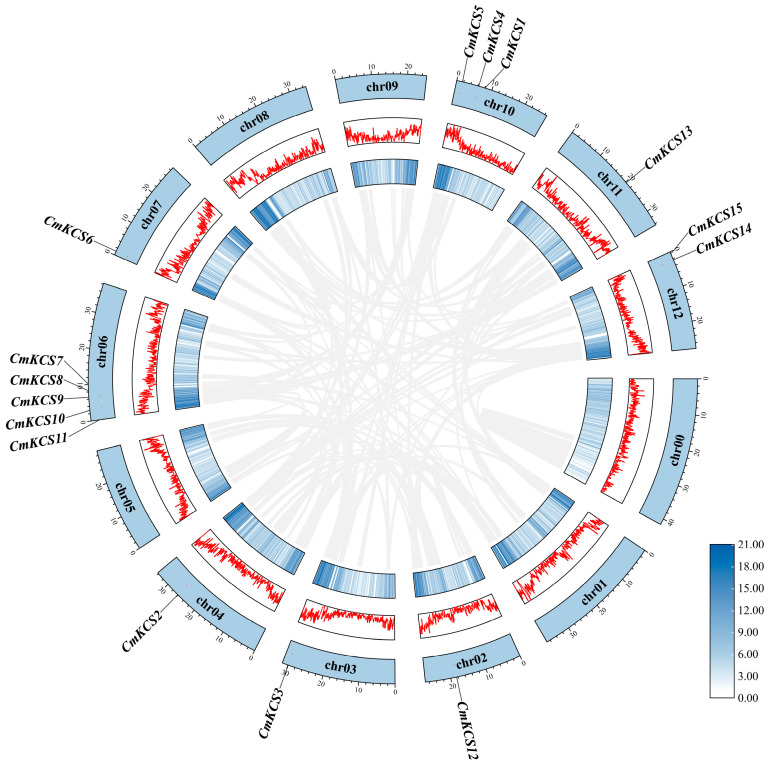
Collinearity analysis of *KCSs* in melon.

**Figure 5:**
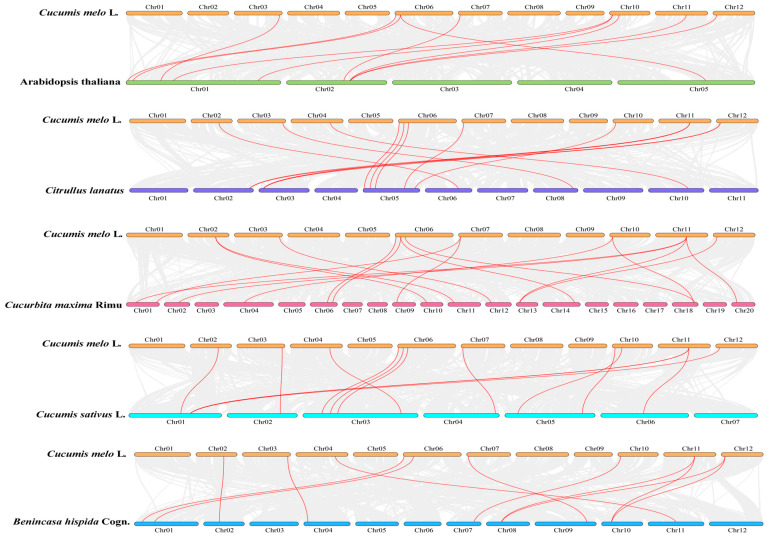
Collinearity analysis of *KCSs* of 7 species.

Two pairs of tandem repeat genes were identified in the *CmKCS* family, namely *CmKCS7* and *CmKCS8*, *CmKCS14* and *CmKCS15*, and no gene duplication phenomenon was found (Figure 4). *CmKCS* may amplify family members through gene tandem repeats during evolution. The analysis of interspecific evolutionary relationships of *KCS* revealed 10 pairs of collinearity between 7 *CmKCS* and 8 *Arabidopsis KCS*, 13 pairs of collinearity between 10 *CmKCS* and 11 watermelon *KCS*, 13 pairs of collinearity between 11 *CmKCS* and 11 cucumber *KCS*, 16 pairs of collinearity between 9 *CmKCS* and 16 Indian pumpkin *KCS*, and 11 pairs of collinearity between 9 *CmKCS* and 9 winter melon *KCS* (Figure 5). *CmKCS3*, *CmKCS5*, *CmKCS6*, *CmKCS11*, *CmKCS13*, and *CmKCS15* all exhibited collinear relationships with different species. There were at least two pairs of collinear relationships between *CmKCS13* and different *Cucurbitaceae* crops, indicating high conservation in the evolutionary process.

### 3.6. Cis-Acting Elements of CmKCS Family Promoter

**Figure 6:**
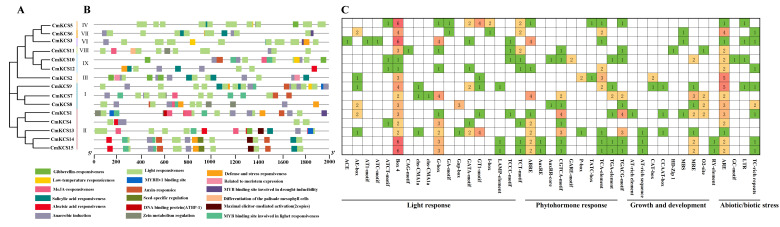
Phylogenetic relationships (**A**), the site (**B**), and number (**C**) of Cis-acting elements in melon *KCSs* promoter.

After removing the CAAT box and TATA box promoters, a total of 41 homeotropic elements were detected in 15 *CmKCS*, mainly including light response, plant hormone response, growth and development, and biotic/abiotic stress response (Figure 6). The number of photoresponsive elements was the highest, with a total of 18 types, among which Box4 elements were common to all members of the *CmKCS* family, with a relatively large quantity. There were 10 types of hormone-responsive elements, with 9 different *CmKCS* members responding to abscisic acid, methyl jasmonate, and salicylic acid, respectively. Among them, *CmKCS13*, *CmKCS14*, and *CmKCS15* respond to three components simultaneously, while the other members showed significant differences in the number of response components. There were a total of 9 types of growth and development response elements, with 8 members possessing MYB binding sites that respond to light reactions, significantly more than other elements. There were four types of stress response elements, with 15 family members containing anaerobic induction response elements, 7 members containing defense and pressure response elements, and the lowest being low-temperature induction response elements. *CmKCS3*, *CmKCS9*, and *CmKCS10* respond to the three stress elements mentioned above and may be involved in the response to multiple stresses.

### 3.7. Expression Analysis of CmKCSs in Three Abiotic Stress Environments

**Figure 7:**
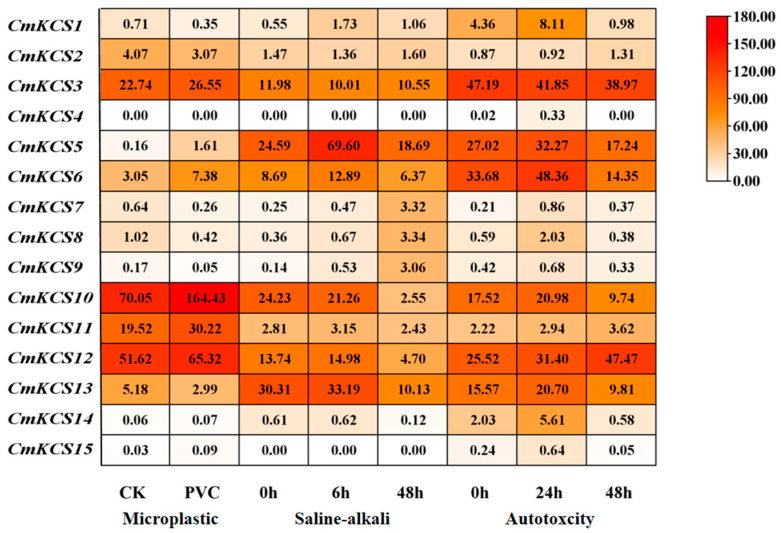
Differential expression of *KCSs* in melon under three abiotic stresses.

The prediction results of promoter cis-acting elements indicated that *CmKCS* may be widely involved in the response process of melon to abiotic stress. To verify this speculation, the differential expression of *CmKCS* members in three abiotic stress environments of autotoxicity, saline-alkali stress, and microplastic exposure environments was analyzed using RNA-seq, and the results are shown in Figure 7. The expression patterns of most *CmKCS* family members under autotoxicity showed an overall trend of first increasing and then decreasing, with most reaching their peak at 24 h. Among them, the expression levels of *CmKCS5*, *CmKCS3*, *CmKCS5*, *CmKCS6*, *CmKCS10*, *CmKCS12*, and *CmKCS13* were relatively high at different stages. *CmKCS12* showed a sustained upregulation trend under stress, reaching its maximum value at 48 h, significantly higher than other genes during the same period.

In saline-alkali stress, the expression levels of *CmKCS1*, *CmKCS5*, *CmKCS6*, *CmKCS11*, *CmKCS12*, and *CmKCS13* all reached their maximum at 6 h and sharply decreased at 48 h. The expression level of *CmKCS5* was significantly higher than other genes at 6 h. The expression levels of *CmKCS7*, *CmKCS8*, and *CmKCS9* reached their maximum at 48 h, reaching 13.56 times, 9.40 times, and 22.65 times higher than at 0 h, respectively. In the microplastics exposure environment, the expression levels of *CmKCS3*, *CmKCS5*, *CmKCS6*, *CmKCS10*, *CmKCS11*, and *CmKCS12* were significantly upregulated compared to the control group. *CmKCS5* was 9.94 times higher than the control group, while the rest were approximately 1.5–2.5 times higher. *CmKCS2*, *CmKCS8*, and *CmKCS13* were significantly downregulated, accounting for 75.5%, 35.5%, and 57.8% of the control group, respectively. The expression levels of *CmKCS4* and *CmKCS15* were extremely low in all three stress environments.

## 4. Discussion

*KCS* is a key enzyme in the synthesis process of VLCFAs. *KCS* can enhance plant defense against adverse external environments by regulating the extension of fatty acids and the deposition of their derivatives to form a waxy layer and suberin. Fifteen *CmKCS* members identified from the melon genome are unevenly distributed on 8 chromosomes, with slightly fewer family members compared to *Arabidopsis* [17], similar in number to cucumbers and watermelons in the *Cucurbitaceae* family. Gene replication is an important way for gene families to produce new members with innovative functions in order to enhance the adaptability of organisms to different environments. The replication mode of the *CmKCS* family is mainly tandem duplication, which may also be the main reason for the uneven distribution of genes on chromosomes. The uneven distribution of *CmKCS* across chromosomes may reflect their functional diversification under environmental pressures. Chromosome 6, with the highest density, suggests potential gene clustering through tandem duplication events, which could enhance functional redundancy or specialization in stress responses. Similarly, chromosomes 10 and 12 may act as genomic hotspots for stress adaptation, allowing coordinated regulation of these genes under adverse conditions. Such chromosomal biases might optimize transcriptional efficiency during abiotic stress challenges, such as autotoxicity or salinity, by grouping functionally related genes in shared regulatory environments.

Subcellular localization predictions showed that the majority of *CmKCS* were localized to the cytoplasmic membrane and chloroplasts, with a small number of members located in vacuoles and cell solutes. This is significantly different from *Arabidopsis*, which is mainly located in the endoplasmic reticulum, but similar to barley [18] and passion fruit [19]. Obviously, there is significant species specificity in the subcellular localization of *KCS*. The organelles responsible for the distribution of *CmKCS* mainly involve lipid synthesis, regulation, and transport and are also the main sites for wax synthesis and secretion. This characteristic may have significant implications for the synthesis, distribution, and deposition of wax and suberin in melons. The cytoplasmic membrane localization of *CmKCS* members aligns with their putative function in synthesizing very-long-chain fatty acids (VLCFAs), which are essential components of cuticular waxes. These waxes act as a physical barrier against water loss and pathogen invasion, a critical adaptation under drought and salinity stress. On the other hand, chloroplast-localized *CmKCS* enzymes may contribute to the remodeling of chloroplast membranes under stress, potentially stabilizing photosynthetic machinery during abiotic challenges such as microplastic toxicity or autotoxicity.

Among the 15 *CmKCS* members, 14 are distributed in the same cluster as cucumber *KCS*. *KCSs* clustered into the same subfamily generally have similar functions [20]. Cucumber and melon originated from the common ancestor *Cucumis prophetarum*, and there are a large number of homologous sequences between the two species in specific genomic regions, with high conservation and very similar functions [21]. The results of the gene collinearity analysis obtained in this study also confirm the above conclusion. Compared to *Arabidopsis*, the I subfamily is unique to the *Cucurbitaceae* plant. Other subfamilies in *Arabidopsis* and *Cucurbitaceae* exhibit relatively uniform distribution, with small differentiation differences and high conservation. Genes with more collinear relationships also show similar distribution characteristics. The *KCS* of *Arabidopsis,* which are clustered in the VI subfamily, are widely involved in the synthesis of epidermal wax, suberin, and phospholipids [22]. Members distributed in the IX subfamily are closely related to the biosynthesis of root epidermal wax layer and suberin, as well as their response to osmotic stress [23]. The corresponding members of the *CmKCS* family may also have similar functions.

The presence of introns and exons in gene structure provides an important basis for determining gene variation, function, and expression. Introns are particularly important, and their quantity and length often have a negative correlation with gene expression [24]. There are 9 members in the *CmKCS* family that do not have introns, accounting for 60%. A similar phenomenon is also observed in barley, where 25 out of 33 barley *KCS* members do not contain introns, accounting for 75.67% [18]. The evolutionary mechanism of intron-free genes has always been controversial. The “introns-early hypothesis” suggests that the absence of introns originated from gene breakage leading to intron loss, while the “introns-late hypothesis” emphasizes that ancestral genes did not have introns. In a study of 33 families of intron-free and intron-scarce genes, it was found that intron-free genes first appeared in primary terrestrial plants, while intron-scarce genes first appeared in green algae. Under stress, intron-free genes perform more functions than intron-rich genes and may play a more important role [25]. The evolutionary pattern of the *CmKCS* family obtained in this study is more in line with the “introns-late hypothesis”. According to the phylogenetic tree results, *CmKCS7*, *CmKCS8*, and *CmKCS9* (subfamily I) formed later (Figure 2), and all three genes are intronless genes (Figure 3). Additionally, *CmKCS9* has more stress response elements, suggesting that intron-free genes appeared later in the evolution of *CmKCS* and played more important roles under adverse conditions.

Homeotropic elements play a crucial role in signal transduction and gene transcription regulation [26], and identifying promoter homeotropic elements can better understand the regulatory mechanisms of gene expression. In this study, it was found that the *CmKCS* promoter sequence contains multiple plant hormones and stress response elements (Figure 6). All members contain at least 2 plant hormone elements and 1 stress response element. Most members have abscisic acid, methyl jasmonate, and salicylic acid hormone-responsive elements. Salicylic acid and abscisic acid activate transcription factors and resistance genes by regulating ROS levels under biotic and abiotic stress, while methyl jasmonate enhances plant defense mechanisms by promoting the synthesis of specific secondary metabolites in plants [27,28]. All members possess anaerobic inducible response elements, which are crucial for the transition of plants from anaerobic to aerobic life and can activate the efficient expression of relevant genes in anaerobic environments. The *CmKCS* may promote secondary metabolite synthesis to cope with different stresses by regulating the expression of related genes in the aforementioned metabolic processes.

*KCS* is widely involved in the establishment of plant response and adaptation to adversity [29]. For example, under drought stress, half of the barley *KCS* are upregulated, promoting VLCFA synthesis and providing important precursor substances for increasing epidermal wax [18]. The *CsKCS6* of Citrus sinensis was significantly induced under drought, saline-alkali, and abscisic acid stress. The ectopic expression of *CsKCS6* significantly increased the VLCFAs content in leaves and stems, and the plant’s drought tolerance and saline-alkali resistance were significantly improved [30]. In this study, most *CmKCS* showed significant differential expression in three different stress conditions. Among them, *CmKCS5*, *CmKCS6*, *CmKCS10*, and *CmKCS12* genes have high expression levels in different stress environments, which may be of great significance in the melon’s response to abiotic stress.

While this study provides valuable insights into the *CmKCS* family and its potential roles in melon stress responses, some limitations should be noted. The findings are primarily based on bioinformatics predictions and RNA-seq data, which require further experimental validation, such as functional characterization of key *CmKCS* through genetic engineering approaches. Additionally, the study focused on a single melon cultivar, and future research could expand to include diverse cultivars to explore genetic variations associated with stress tolerance. Further investigations into the regulatory networks and multi-omics integration would also deepen our understanding of the molecular mechanisms underlying melon stress adaptation.

## 5. Conclusions

In this study, 15 *CmKCS* were identified and characterized in the melon genome, revealing their phylogenetic relationships, gene structures, and expression patterns under various stress conditions. The novelty of this study lies in the comprehensive genome-wide analysis of the *CmKCS* family, which has not been previously reported in melon. The *CmKCS* members were divided into nine subfamilies, with those in subfamilies I, IV, and VI exhibiting conserved domains and undergoing sequence mutations, leading to family expansion through tandem repeats. Additionally, multiple hormone and stress response elements were identified in the promoter regions of *CmKCS*, with abscisic acid, methyl jasmonate, salicylic acid, and anaerobic induction elements being the most abundant. Notably, the expression analysis revealed that *CmKCS5*, *CmKCS6*, *CmKCS10*, and *CmKCS12* are highly responsive to autotoxicity, saline-alkali stress, and microplastic exposure, suggesting their potential roles in melon’s stress adaptation mechanisms. These findings provide new insights into the functional diversification of the *CmKCS* family and their contributions to stress resistance in melon. Future investigations should focus on the functional validation of key *CmKCS* through genetic engineering approaches to elucidate their specific roles in stress responses. Further exploration of the regulatory networks involving *CmKCSs* and their interactions with other metabolic pathways under stress conditions will deepen the understanding of their mechanisms. This study provides a foundation for further research on the molecular mechanisms of stress adaptation in melon and highlights the potential importance of the *CmKCS* family in crop improvement.

## Figures and Tables

**Table 1:** Physicochemical properties of *KCSs* in melon.

Gene Name	Gene Accession No.	Chromosome Location	Number of Amino Acids	Molecular Weight	Isoelectric Point	Instability Index	Subcellular Localization
Cm*KCS1*	MELO3C023819	chr10: 6293610 .. 6295277 (+)	469	52,909.63	8.89	39.44	Chloroplast
Cm*KCS2*	MELO3C026618	chr04: 26126012 .. 26127500 (+)	450	50,104.13	9.10	28.74	Vacuole
Cm*KCS3*	MELO3C010941	chr03: 29913624 .. 29915701 (+)	514	57,383.45	9.10	38.66	Plasma membrane
Cm*KCS4*	MELO3C023816	chr10: 6249025 .. 6251555 (−)	479	54,214.25	9.00	30.37	Chloroplast
Cm*KCS5*	MELO3C012246	chr10: 1721492 .. 1722982 (−)	496	56,076.90	9.24	38.90	Plasma membrane
Cm*KCS6*	MELO3C016930	chr07: 1070353 .. 1074857 (−)	294	33,861.82	9.42	39.75	Chloroplast
Cm*KCS7*	MELO3C006850	chr06: 6592418 .. 6593839 (+)	437	52,932.23	7.23	47.98	Plasma membrane
Cm*KCS8*	MELO3C006849	chr06: 6584354 .. 6586197 (+)	490	55,396.60	8.82	42.74	Vacuole
Cm*KCS9*	MELO3C006847	chr06: 6577077 .. 6578900 (+)	483	54,014.79	8.18	46.00	Plasma membrane
Cm*KCS10*	MELO3C006484	chr06: 3594920 .. 3597581 (+)	502	56,832.27	9.23	35.21	Plasma membrane
Cm*KCS11*	MELO3C005986	chr06: 395141 .. 396958 (−)	514	57,502.64	8.72	39.29	Plasma membrane
Cm*KCS12*	MELO3C026623	chr02: 18370129 .. 18372901 (−)	588	66,670.98	9.29	42.39	Plasma membrane
Cm*KCS13*	MELO3C013669	chr11: 20326893 .. 20328793 (−)	461	52,238.29	8.37	37.51	Cytosol
Cm*KCS14*	MELO3C020670	chr12: 2355748 .. 2358649 (+)	462	52,016.52	9.10	34.42	Chloroplast
Cm*KCS15*	MELO3C020668	chr12: 2336969 .. 2339666 (+)	462	52,258.82	9.06	35.80	Chloroplast

## Data Availability

The datasets generated and analyzed during the current study will be available from the corresponding author upon reasonable request.
